# Electroacupuncture at ST37 Enhances Jejunal Motility via Excitation of the Parasympathetic System in Rats and Mice

**DOI:** 10.1155/2016/3840230

**Published:** 2016-10-12

**Authors:** Mengqian Yuan, Yuqin Li, Yidan Wang, Na Zhang, XuanMing Hu, Yin Yin, Bing Zhu, Zhi Yu, Bin Xu

**Affiliations:** ^1^Key Laboratory of Integrated Acupuncture and Drugs, Nanjing University of Chinese Medicine, Ministry of Education, 138 Xianlin Road, Qixia District, Nanjing, Jiangsu 210029, China; ^2^Institute of Acupuncture and Moxibustion, China Academy of Chinese Medical Sciences, Beijing, China

## Abstract

*Background.* The roles of the sympathetic and parasympathetic systems in mediating the effect of electroacupuncture (EA) at ST37 on jejunal motility have yet to be demonstrated.* Aim*. We used rats and mice to investigate the effect and mechanism of action of EA at ST37 on jejunal motility.* Methods.* Jejunal motility was recorded by a balloon placed in the jejunum and connected to a biological signal collection system through a transducer. The effects of EA (3 mA) at ST37 were evaluated in Sprague-Dawley rats without drugs and with the administration of clenbuterol, propranolol, acetylcholine, and atropine. Further, the efficacy of EA at different intensities (1/2/4/6/8 mA) was measured in wild-type mice and *β*_1_ *β*_2_^−/−^ mice and M_2_M_3_^−/−^ mice.* Results.* In Sprague-Dawley rats, the excitatory effect of EA at ST37 on jejunal motility disappeared in the presence of the muscarinic receptor antagonist atropine. EA at ST37 was less effective in M_2_M_3_^−/−^ mice than in wild-type mice. Furthermore, to a certain extent, there existed “intensity-response” relationship between jejunal motility and EA.* Conclusions.* EA at ST37 can enhance jejunal motility in rats and mice mainly via excitation of the parasympathetic pathway. There is an “intensity-response” relationship between EA and effect on jejunal motility.

## 1. Introduction

Acupuncture is a traditional Chinese medicine (TCM) therapeutic technique in which sharp, thin needles are inserted into specific points on the body to restore homeostatic balance [1]. The doctrine of acupuncture has been known since 400 BC [2] and, over the course of thousands of years, it has been applied in China extensively to cure various diseases [3]. A World Health Organization study showed that, at present, acupuncture is being used in 183 of 202 surveyed countries [4].

Disorder of gastrointestinal motility is a common problem that is seen in a range of diseases, such as constipation, diarrhea, chronic intestinal pseudoobstruction, and irritable bowel syndrome (IBS) [5, 6]. Acupuncture possesses credible therapeutic efficacy on gastrointestinal dysfunction [7, 8], but the underlying mechanism remains unclear.

The autonomic nervous system is closely involved in the modulation of visceral function [9, 10]. Several studies have shown that acupuncture stimulation regulates gastrointestinal motility primarily via autonomic nervous reflexes [11–13]. In animal models, acupuncture at the abdominal skin inhibits gastrointestinal motility mainly via the sympathetic pathway [14], whereas acupuncture at a hind paw enhances gastric motility mainly via the parasympathetic pathway [15, 16]. However, the evidence for this is still scarce.

Electroacupuncture (EA) is a quantifiable treatment because the stimulation frequency and intensity can be controlled [17], and, therefore, it is widely used in clinical and experimental research. Previous studies have suggested that different frequencies of EA can induce different peripheral reactions [18, 19], but little effort has been made to investigate how EA with different intensities affects jejunal motility.

We hypothesized that the immediate effect of EA at the hind paw of rats and mice (ST37) on jejunal motility may mostly be by excitation of the parasympathetic pathway and inhibition of the sympathetic pathway. To test this hypothesis, we performed adrenoceptors antagonist and agonist to determine the role of sympathetic pathway as well as muscarinic receptors antagonist and agonist for parasympathetic pathway in normal rats, and gene knockout mice are employed to do a further validation. We also investigated how change in intensity of EA affected jejunal motility. Overall, our data revealed that EA at ST37 enhances jejunal motility mainly via excitation of the parasympathetic pathway. Furthermore, to a certhain degree, there existed an “intensity-response” pattern in EA stimulation.

## 2. Materials and Methods

### 2.1. Animals

Male Sprague-Dawley rats (180–230 g, aged 12–14 weeks) were obtained from the Model Animal Research Center of Nanjing Medical University, China. The mice used in the study were all males, weighing 22–28 g and aged 8–12 weeks; *β*_1_ *β*_2_^−/−^ mice (Adrb1^tmlBkk^Adrb2^tmlBkk^/J, J003810) were donated by the Jackson Laboratory, USA; M_2_M_3_^−/−^ mice (D2; 129-Chrm2^tml^  Chrm3^tml^, D0407) were obtained from Kumamoto University, Japan. The wild-type (WT) mice were purchased from the Model Animal Research Center of Nanjing University, China. All animals were housed at an ambient temperature of 22°C and relative humidity of 40%–60% at the Experimental Animal Center, Nanjing University of TCM, where the light/dark cycle was 12/12 h. The feed adaptation lasted for 7 days. All animal experimental procedures were performed according to the* Guide for the Care and Use of Laboratory Animals* (National Research Council, Washington, DC).

### 2.2. Drugs

Rats and mice were anesthetized by urethane (U2500; Sigma-Aldrich, St. Louis, MO, USA). The beta-adrenoceptor agonist clenbuterol hydrochloride (C5423), beta-adrenoceptor antagonist propranolol hydrochloride (P0084), muscarinic receptor antagonist atropine hydrochloride (A6625), and muscarinic receptor agonist acetylcholine hydrochloride (A0132) were all supplied by Sigma-Aldrich, St. Louis, MO, USA. The concentration, dosages, and route of administration of the drugs were as follows: (1) urethane: 20%, 8 mL/kg for rats and 5 mL/kg for mice, intraperitoneally; (2) clenbuterol: 0.2%, maintenance dose: 80 *μ*L·min^−1^·kg^−1^, intravenously; (3) propranolol: 0.4%, initial dose: 1.0 mL·kg^−1^, maintenance dose: 40 *μ*L·min^−1^·kg^−1^, intravenously; (4) acetylcholine: 0.1%, maintenance dose: 20 *μ*L·min^−1^·kg^−1^, intravenously; and (5) atropine: 0.2%, initial dose: 0.8 mL·kg^−1^, maintenance dose: 40 *μ*L·min^−1^·kg^−1^, intravenously.

### 2.3. Recording of Jejunal Motility

A small incision (length: 5–8 mm in rats and 2-3 mm in mice) was made below the xiphoid and a small balloon (diameter ~2 mm) made of flexible condom rubber was placed in the jejunum, about 3–5 cm (rats) or 0.8–1.2 cm (mice) downstream from the suspensory ligament of the duodenum. The balloon was filled with warm water and connected via a polyethylene tube of ~10-cm length to a transducer (YPJ01; Chengdu Instrument Factory, China); the signal was collected with a biological signal-sampling system (RM6240; Chengdu Instrument Factory) for analysis.

### 2.4. Experimental Procedure of EA at ST37 in Rats without and with Drugs

**Figure 1:**
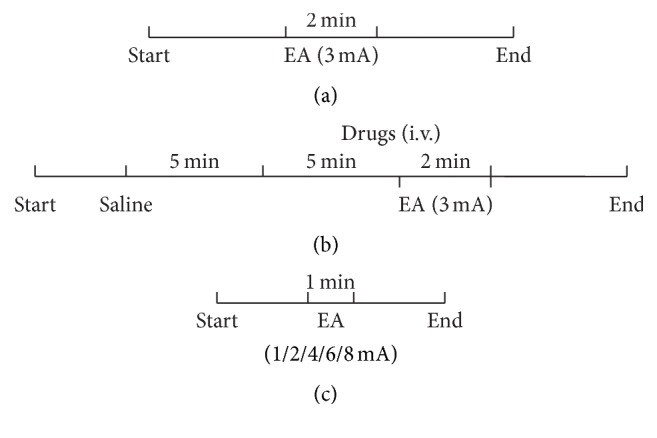
The experimental procedure. (a) Timeline of intervention in the nondrug treated rats. (b) Timeline of intervention in the drug treated rats. (c) Timeline of intervention in mice with different intensities of electroacupuncture (1 mA, 2 mA, 4 mA, 6 mA, and 8 mA).

The rats were divided into five groups with 8 rats per group: (1) control group (no drug administration), (2) the clenbuterol group, (3) the propranolol group, (4) the acetylcholine group, and (5) the atropine group. All rats were fasted for 12 hours, with free access to water, prior to being anesthetized with urethane. After anesthesia, rats in the 4 drug treated groups underwent endotracheal intubation and cannulation of the left internal jugular vein (for drug administration); the control group rats were not intubated or cannulated. The balloon was then inserted into the jejunum of the rats as described earlier. The experiment procedures of the rats in control group and the four drug groups are shown in Figures 1(a) and 1(b), respectively. The intensity of EA in all 5 groups was 3 mA and lasted for 2 min. Before application of EA, we ensured that the baseline jejunal pressure was between 0.28 and 0.32 kPa in all rats. During the experiment, the mice were placed on an electric heating board to maintain body temperature at 37°C ± 0.5°C.

### 2.5. Experimental Procedure of EA at ST37 with Different Intensities in WT, *β*_1_ *β*_2_^−/−^, and M_2_M_3_^−/−^ Mice

We also observed the effect of EA at different intensities in WT, *β*_1_ *β*_2_^−/−^, and M_2_M_3_^−/−^ mice. All mice were fasted for 4 hours, with free access to water, prior to being anesthetized with urethane. After anesthesia and placement of the jejunal balloon, we first ensured that the jejunal pressure was stabilized between 0.28 and 0.32 kPa. Then, EA at different intensities (1 mA, 2 mA, 4 mA, 6 mA, and 8 mA) was applied for 1 minute each. A new stimulus was applied only after the jejunal pressure had recovered to the baseline. The experiment flow in the mice is shown in Figure 1(c).

### 2.6. EA Stimulation

ST37 (Shangjuxu) is located 5 mm below the knee joint and 1 mm lateral to the margo anterior tibiae in rats and 2 mm below the knee joint and 0.5 mm lateral to the margo anterior tibiae in mice. A pair of stainless steel acupuncture needles (diameter: 0.3 mm) were inserted to approximately 3 mm depth at the right ST37. The needles were connected to a Han electroacupuncture therapeutic stimulator (LH_4_02A; Beijing Huawei Industrial Development Corporation, China). The frequency setting for EA was 1/15 Hz.

### 2.7. Assessment

Jejunal pressure during EA (dur-EA) was compared with the pressure before EA (pre-EA) [20]. Change in jejunal pressure of >105% (Equation (1)) was taken as evidence of enhanced jejunal motility

(1) $$\begin{array}{c} \text{Percentage change}=\frac{\text{dur-}EA}{\text{pre-}EA}\times 100\%. \end{array}$$

### 2.8. Statistical Analysis

Data were analyzed by using SPSS 18.0 (SPSS Inc., Chicago, IL, USA) and GraphPad Prism 5.0 (GraphPad Software, CA, USA). Data were expressed as means ± SEM (the standard error of the mean). The paired-sample *t*-test was used for comparisons within groups and the independent-samples *t*-test for comparisons between groups. *P* < 0.05 was considered statistically significant. The data curve of different intensity was fitted with (2). *X* is log of intensity. *Y* is response, increasing as *X* increases. Top and bottom are plateaus in the same units as *Y*. log EC_50_ is same log units as *X*:

(2) $$\begin{array}{c} Y=\text{Bottom}+\frac{\left(\text{Top-Bottom}\right)}{1+10^{X\text{-}log\;{EC}_{50}}}. \end{array}$$

## 3. Results

### 3.1. Effect of EA at ST37 on Jejunal Motility in Nondrug Treated Rats

**Figure 2:**
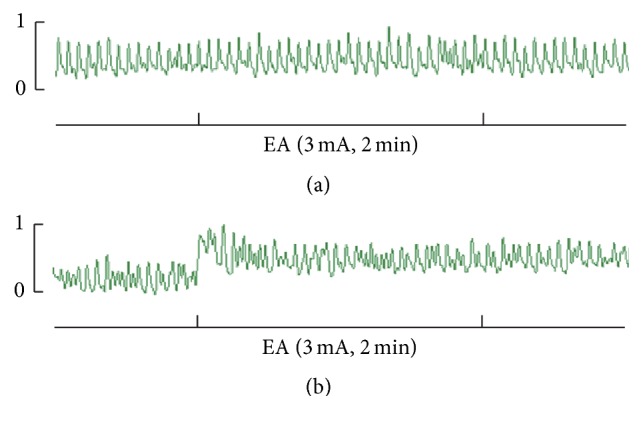
Jejunal motility before and after EA at ST37. (a) A representative trace of jejunal motility in a rat before EA treatment. (b) A representative trace of jejunal motility enhanced by EA at ST37.

**Figure 3:**
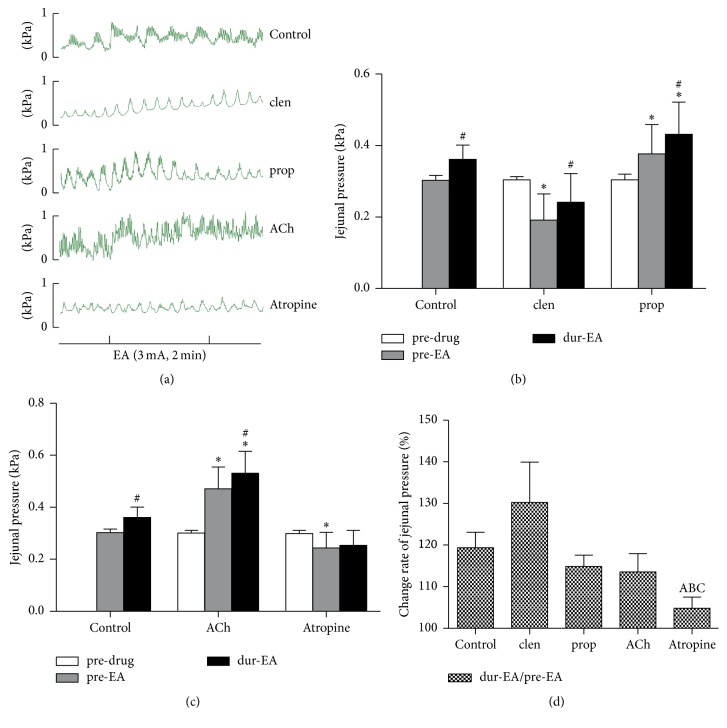
Jejunal motility in response to EA at ST37 in the presence of drugs in rats. (a) Representative traces of jejunal motility regulated by EA without and with the administration of propranolol, clenbuterol, acetylcholine, or atropine. (b) Changes in jejunal pressure were detected before drug administration, before EA, and during EA in the control group, propranolol group, and clenbuterol group. Propranolol promoted jejunal pressure significantly, and clenbuterol inhibited jejunal pressure significantly. EA at ST37 promoted jejunal pressure in all the groups significantly. (c) Changes in jejunal pressure were detected before drug administration, before EA, and during EA in the control group, acetylcholine group, and atropine group. Acetylcholine promoted jejunal pressure significantly and atropine inhibited jejunal pressure significantly. EA at ST37 enhanced jejunal pressure in the acetylcholine group, but it had no effect in the atropine group. Data are expressed as mean ± SEM (*n* = 8 rats at each time period per group). ^*∗*^ *P* < 0.05 versus pre-drug value; ^#^ *P* < 0.05 versus pre-EA value; paired* t*-test. (d) Percentage increased in jejunal pressure following EA in all five experimental groups. Promotion of jejunal motility with EA is significantly lower in the atropine group than in the other groups. Data are expressed as mean ± SEM (*n* = 8 rats). ^A^ *P* < 0.05 versus control group, ^B^ *P* < 0.05 versus clenbuterol group and ^C^ *P* < 0.05 versus propranolol group; independent-samples* t*-test. EA: electroacupuncture; prop: propranolol; clen: clenbuterol; Ach: acetylcholine.

The jejunal baseline pressure was maintained at approximately 0.3 kPa before EA in all rats (Figure 2(a)). EA at ST37 caused significant increase in jejunal motility, as indicated by the rise in pressure from 0.30 ± 0.02 kPa to 0.36 ± 0.04 kPa (*P* < 0.01; Figures 2(b) and 3(b)).

### 3.2. Effects of EA at ST37 on Jejunal Motility in Drug Treated Normal Rats

First, we explored the role of sympathetic pathway in the effect of EA at ST37. As shown in Figures 3(a) and 3(b), administration of the beta agonist clenbuterol markedly decreased the pressure in the jejunum (from 0.30 ± 0.01 kPa to 0.19 ± 0.07 kPa; *P* < 0.05); conversely, administration of the beta blocker propranolol significantly increased the pressure (from 0.38 ± 0.08 kPa to 0.43 ± 0.09 kPa; *P* < 0.05). Following EA at ST37 in the presence of clenbuterol, jejunal pressure increased significantly from 0.19 ± 0.07 kPa pre-EA to 0.24 ± 0.08 kPa dur-EA (*P* < 0.01) and, in the presence of propranolol, jejunal pressure increased from 0.38 ± 0.08 kPa pre-EA to 0.43 ± 0.09 kPa dur-EA. This suggests that the sympathetic pathway may not play an important role in the effect of EA at ST37 at the intensity of 3 mA.

We also examined the role of the parasympathetic pathway. As shown in Figures 3(a) and 3(c), administration of acetylcholine significantly increased the pressure in the jejunum from 0.30 ± 0.01 kPa to 0.47 ± 0.08 kPa (*P* < 0.05), and the administration of atropine significantly decreased the pressure from 0.30 ± 0.01 kPa to 0.21 ± 0.04 kPa (*P* < 0.05). Then, in the presence of acetylcholine, EA at ST37 still increased jejunal pressure significantly (from 0.47 ± 0.08 kPa pre-EA to 0.53 ± 0.08 kPa dur-EA; *P* < 0.01). However, in the presence of atropine, EA at ST37 had no significant effect on jejunal motility, the pressures recorded being 0.21 ± 0.04 kPa pre-EA versus 0.22 ± 0.04 kPa dur-EA (*P* > 0.05).

Further, we compared the percentage change in jejunal pressure following EA at ST37 in the 5 groups (Figure 3(d)); the change was significantly lower in the atropine group than in the other 4 groups. Taken together, these data suggested that the parasympathetic pathway may mediate the effect of EA at ST37.

### 3.3. Effects of EA at ST37 with Different Intensities on Jejunal Motility in WT Mice

**Figure 4:**
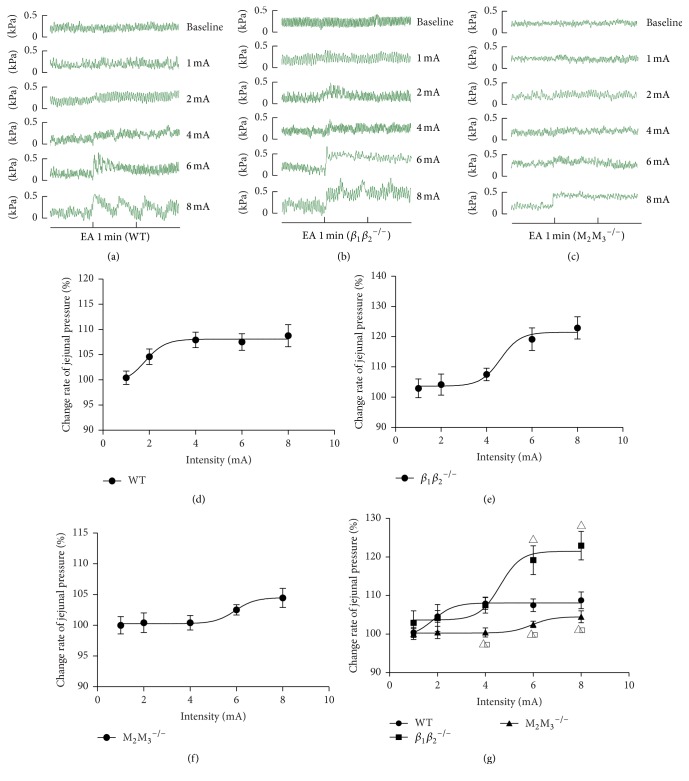
Jejunal motility in response to EA at ST37 with different intensities in WT, *β*_1_ *β*_2_^−/−^, and M_2_M_3_^−/−^ mice. (a–c) Representative traces of jejunal motility with different intensities in WT, *β*_1_ *β*_2_^−/−^, and M_2_M_3_^−/−^ mice. (d–f) The fitting curve of enhanced effect of EA with different intensities on jejunal motility in WT, *β*_1_ *β*_2_^−/−^, and M_2_M_3_^−/−^ mice. The three figures show the “intensity-response” relationships between the different intensities of EA (1/2/4/6/8 mA) and the change in jejunal pressure. (g) The fitting curve of enhanced effect of EA with different intensities on jejunal motility in WT, *β*_1_ *β*_2_^−/−^, and M_2_M_3_^−/−^ mice. Data are expressed as mean ± SEM (*n* = 8 mice). ^△^ *P* < 0.05 versus WT mice and ^□^ *P* < 0.05 versus *β*_1_ *β*_2_^−/−^ mice; independent-samples* t*-test. WT: wild-type.

Following EA at ST37 in WT mice, the percentage change in pressure was >100% at all intensities except at 1 mA (Figures 4(a) and 4(d)). The log EC_50_ value of the EA stimulation in WT mice was 1.82 ± 0.49 mA, and jejunal motility increased with increasing intensity until 4 mA, after which the effect plateaued. In summary, these results suggested that there existed an intensity-response relationship between the intensity of EA and of its effect on jejunal motility.

### 3.4. Effects of EA at ST37 with Different Intensities on Jejunal Motility in *β*_1_ *β*_2_^−/−^ and M_2_M_3_^−/−^ Mice

We used *β*_1_ *β*_2_ adrenoceptor double-knockout mice (*β*_1_ *β*_2_^−/−^) and M_2_M_3_ muscarinic receptor double-knockout mice (M_2_M_3_^−/−^) to further examine the involvement of the autonomic nervous system in the effect of EA at ST37. As shown in Figure 4, the log EC_50_ value of the EA stimulation in WT mice was 1.82 ± 0.49 mA (Figure 4(d)), while the value was 4.61 ± 0.58 mA (Figure 4(e)) and 5.95 ± 0.64 mA (Figure 4(f)) in *β*_1_ *β*_2_^−/−^ mice and M_2_M_3_^−/−^ mice, respectively. Compared with WT mice, the M_2_M_3_^−/−^ mice showed significantly lower percentage change in pressure following EA at ST37 at the EA intensities of 4 mA, 6 mA, and 8 mA and, though not statistically significant, a similar trend was observed at the intensity of 2 mA. However, we found no difference between WT and *β*_1_ *β*_2_^−/−^ mice in the effect of EA at the intensities of 1 mA, 2 mA, and 4 mA (Figure 4(g)). Taken together, these data further confirmed that the effect of EA at ST37 was mediated via the parasympathetic pathway.

## 4. Discussion

In this study we investigated whether the effect of EA at ST37 on jejunal motility was primarily via excitation of the parasympathetic pathway and inhibition of the sympathetic pathway. We used adrenoceptor and muscarinic receptor antagonists and agonists to determine the role of the sympathetic and parasympathetic pathways in normal rats. We verified the findings using WT mice, *β*_1_ *β*_2_^−/−^ mice, and M_2_M_3_^−/−^ mice. The relationship between intensity of EA and the effect on jejunal motility was also studied. Our results showed that EA at ST37 enhanced jejunal motility mainly via excitation of the parasympathetic pathway in rats and mice. Furthermore, we found that, to some extent, the jejunal response was related to the intensity of the stimulation relationship.

As shown in Figure 2, EA at ST37 enhanced jejunal motility obviously; this is in accord with previous reports. Exploration of the underlying neural mechanisms (Figures 3 and 4) showed that the parasympathetic pathway plays a critical role in mediating the effect of EA at ST37; this was demonstrated in two ways. First, in normal rats, the excitatory effect disappeared in the presence of the muscarinic receptor antagonist atropine (Figure 3(c)). Second, EA at ST37 was less effective in M_2_M_3_^−/−^ mice than in WT mice (Figure 4(g)). In contrast, there was no evidence to show that inhibition of the sympathetic pathway was responsible for the enhancement of jejunal motility.

Since at least the 1960s, several studies have demonstrated that gastrointestinal motility can be influenced by a somatic-autonomic reflex [21, 22]. Sato et al. reported that pinching stimulation of the abdominal wall inhibits gastrointestinal motility in anesthetized rats, whereas similar stimulation of a hind paw enhances gastrointestinal motility [9, 23, 24]. Noguchi et al. found the similar results when they investigated the change of gut motility response to electrical or mechanical acupuncture of abdominal wall or hind paws points, and they elucidated the important role of splanchnic inhibitory nerves and vagal excitatory nerves played in the dual directional effects [25, 26].

Zhu et al. also observed the similar acupuncture effect [11, 27]; moreover, they proposed the existence of “homotopic and heterotopic acupoints” to explain the effects and mechanism of action [28]. Homotopic acupoint is in the same spinal cord segment and that of a heterotopic acupoint in a different spinal cord segment, from which the efferent innervates visceral organs; acupuncture at this point could therefore inhibit or facilitate gastrointestinal motility via the sympathetic or parasympathetic pathways [15, 16]. According to this concept, ST25 and ST37 are, respectively, the homotopic and heterotopic acupoints for the jejunum. Consistent with this theory, in our previous study we have shown that EA at ST25 inhibits gastric and jejunal motility via the sympathetic pathway [14, 29] and, in this present study, we have demonstrated the critical role of the parasympathetic pathway in mediating the effect of EA at ST37 on jejunal motility.

It has been well accepted that postganglionic sympathetic terminals release the neurotransmitter adrenaline, which activates beta_1_- and beta_2_-adrenoceptors on the smooth muscle cell surface and eventually leads to smooth muscle relaxations [30]. Correspondingly, parasympathetic terminals release acetylcholine to muscarinic receptors and finally contract the smooth muscle. Although five muscarinic receptor subtypes (M_1–5_) have been recognized in the gastrointestinal tract, the M_2_ and M_3_ muscarinic receptor subtypes are found with preponderance [31]. Thus, we performed beta-adrenoceptors antagonist and agonist to investigate the role of sympathetic pathway as well as muscarinic receptors antagonist and agonist for parasympathetic pathway in rats. What is more, we then used the mutant mice lacking of *β*_1_ *β*_2_ or M_2_M_3_ in our study to make a further validation.

Previous studies have demonstrated that only stimulations that exceed the threshold for activation of A*δ* (or group III) and/or C-fibers (or group IV) could regulate gastric motility significantly [11]. Koizumi et al. proved that activation of group III fibers in the sural nerve of the hind limb could increase jejunal motility and that maximal increase was obtained when the stimulus intensity was sufficient to activate group IV afferent fibers [24]. In our study, jejunal motility showed almost no response to EA at 1 mA in WT mice, and it plateaued when the intensity was above 4 mA. We found that EA at 2 mA (the log EC_50_ value of EA stimulation was 1.82 ± 0.49 mA) could activate A*δ* fibers and EA at 4 mA could activate C fibers.

Based on presently available evidence, we could speculate that the neural circuit of EA at ST37 affects jejunal motility. The A*δ* fibers and/or C fibers in the nerves of the hind paw are activated by EA at ST37, and they convey the excitatory signals to supraspinal centers. After a series of integrations in these centers, the parasympathetic pathway is activated; the nerve terminals release the neurotransmitter acetylcholine, which exerts excitatory effects on jejunal smooth muscle.

## 5. Conclusions

In conclusion, we have clearly demonstrated the critical role of the parasympathetic pathway in the promotion of jejunal motility in response to EA at ST37 in rats and mice. We have also demonstrated the existence of an intensity-response relationship between EA and jejunal motility, with greater intensity of EA at ST37 producing greater increase in jejunal motility, at least up to a certain point. Our study adds to the accumulating evidence that acupuncture regulates gastrointestinal function via a somatic-autonomic reflex. The next step is to explore the crosstalk of sympathetic and parasympathetic response to the compatibility of two or more acupoints.
